# Supplementary material for: Metabolic activities are selective modulators for individual segmentation clock processes
Source: Nat Commun. 2025 Jan 20;16:845. doi: 10.1038/s41467-025-56120-5 (PMC11746943; doi:10.1038/s41467-025-56120-5)
Supplement: Supplementary file 1 — Supplementary Information [file 41467_2025_56120_MOESM1_ESM.pdf]

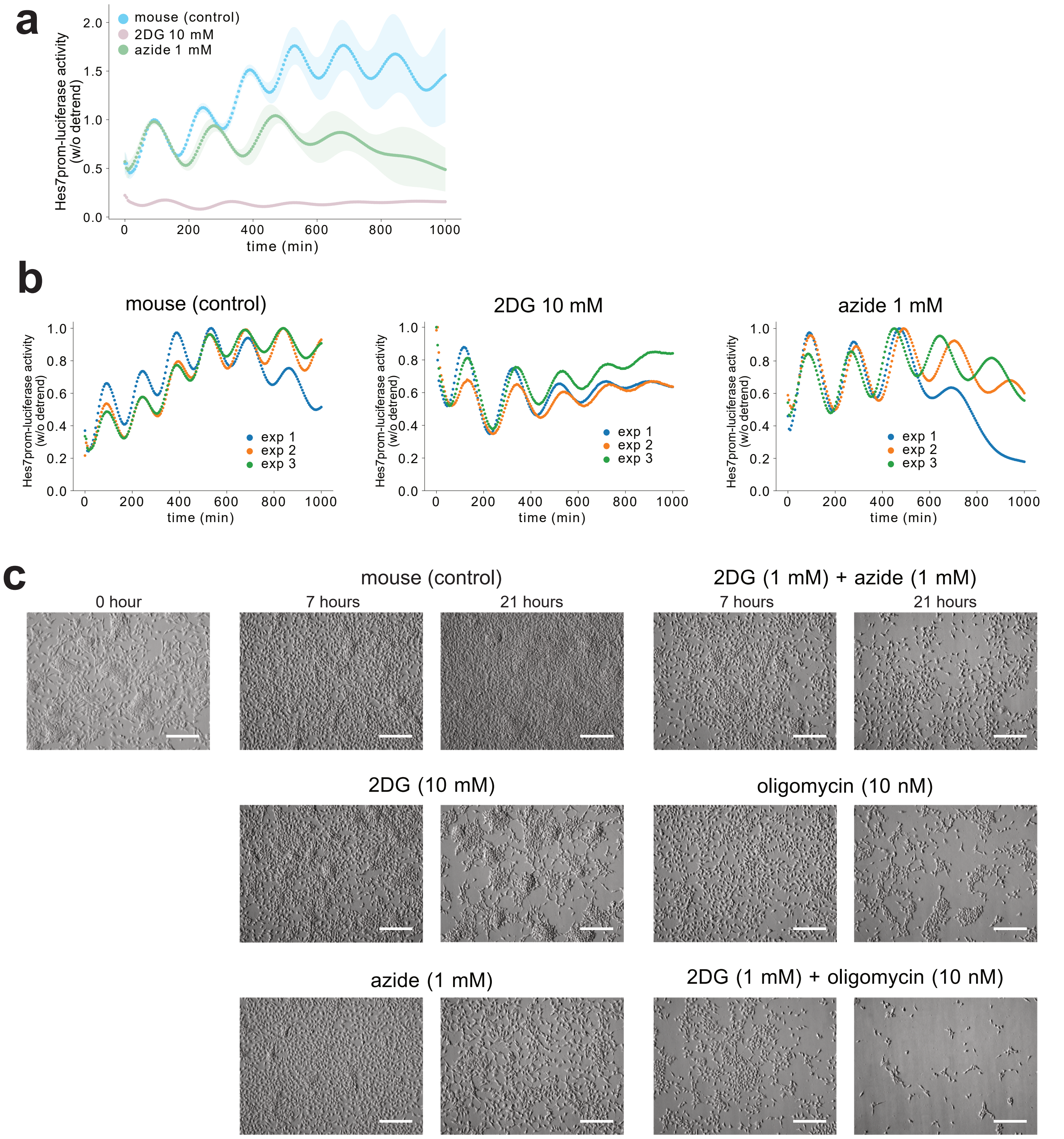

Supplementary Figure 1 Effects of metabolic inhibitions on oscillatory signals and cell survival

a, Raw data of the oscillatory signals shown in Fig. 2b. Shading indicates mean ± sd (n = 3). The first peak of the oscillatory signal of the control sample was set to 1. **b,** Individual tracks of a from 3 independent experiments. The maximum value was set to 1. **c,** Bright-field images of mouse PSM cells treated with metabolic inhibitors. Representative images from 3 independent experiments. Scale bars: 200 µm. Source data are provided as a Source Data file.

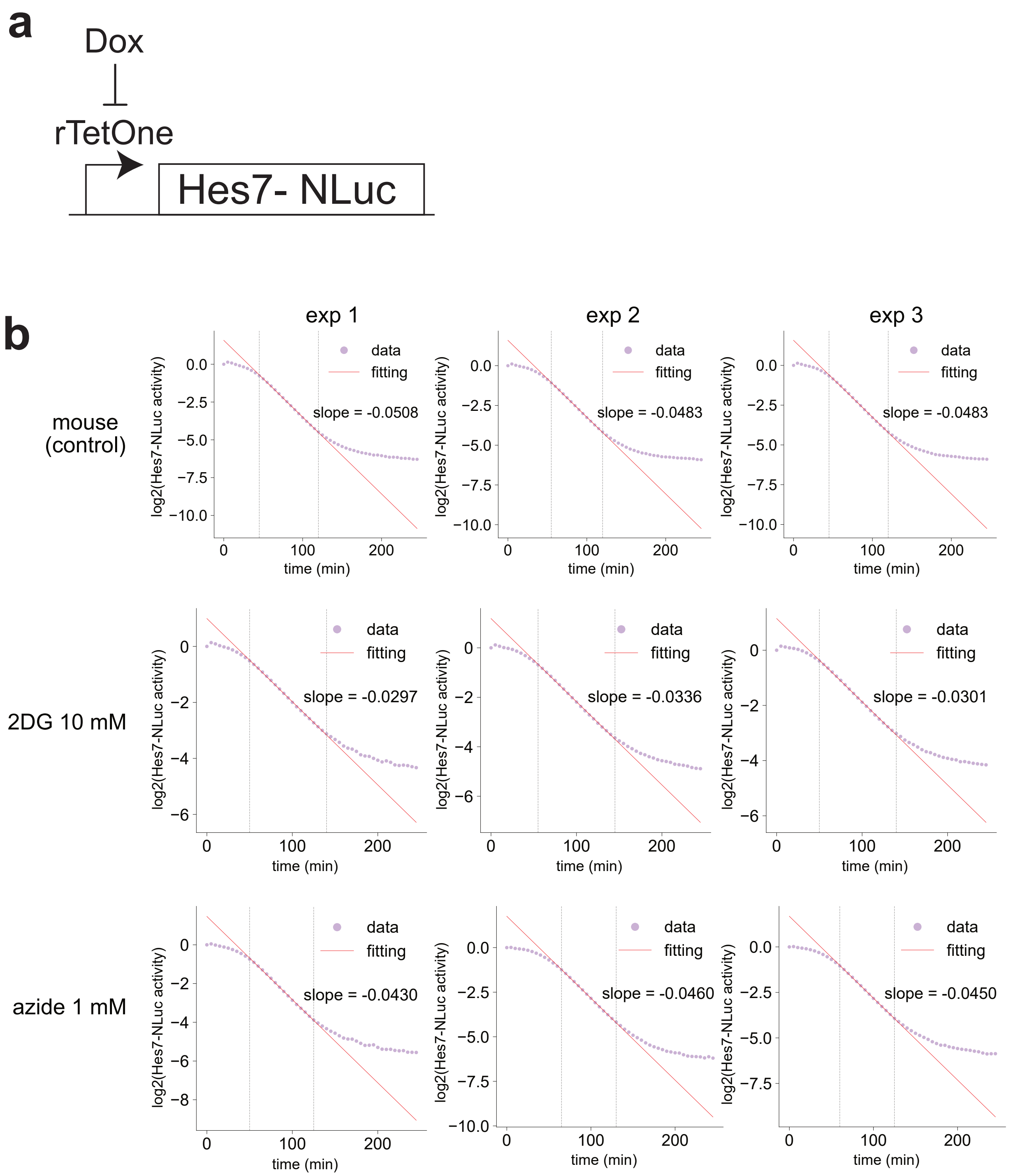

Supplementary Figure 2 Protein degradation assay

a, Schematic representation of the rTetOne promoter-Hes7-NLuc reporter. Upon Dox addition at time 0, rTetOne promoter is repressed, and the transcription of Hes7-NLuc is halted. By monitoring the decay of the Hes7-NLuc signal, the protein degradation rate is estimated. **b,** Raw data and fitting of the Hes7 protein degradation assay shown in Fig. 2e. Dashed lines indicate the most linear region considered by the RANSAC algorithm for the fitting. Slope of the fitted line is shown, and it was converted to the half-life using the equation: half-life = -1/slope. Source data are provided as a Source Data file.

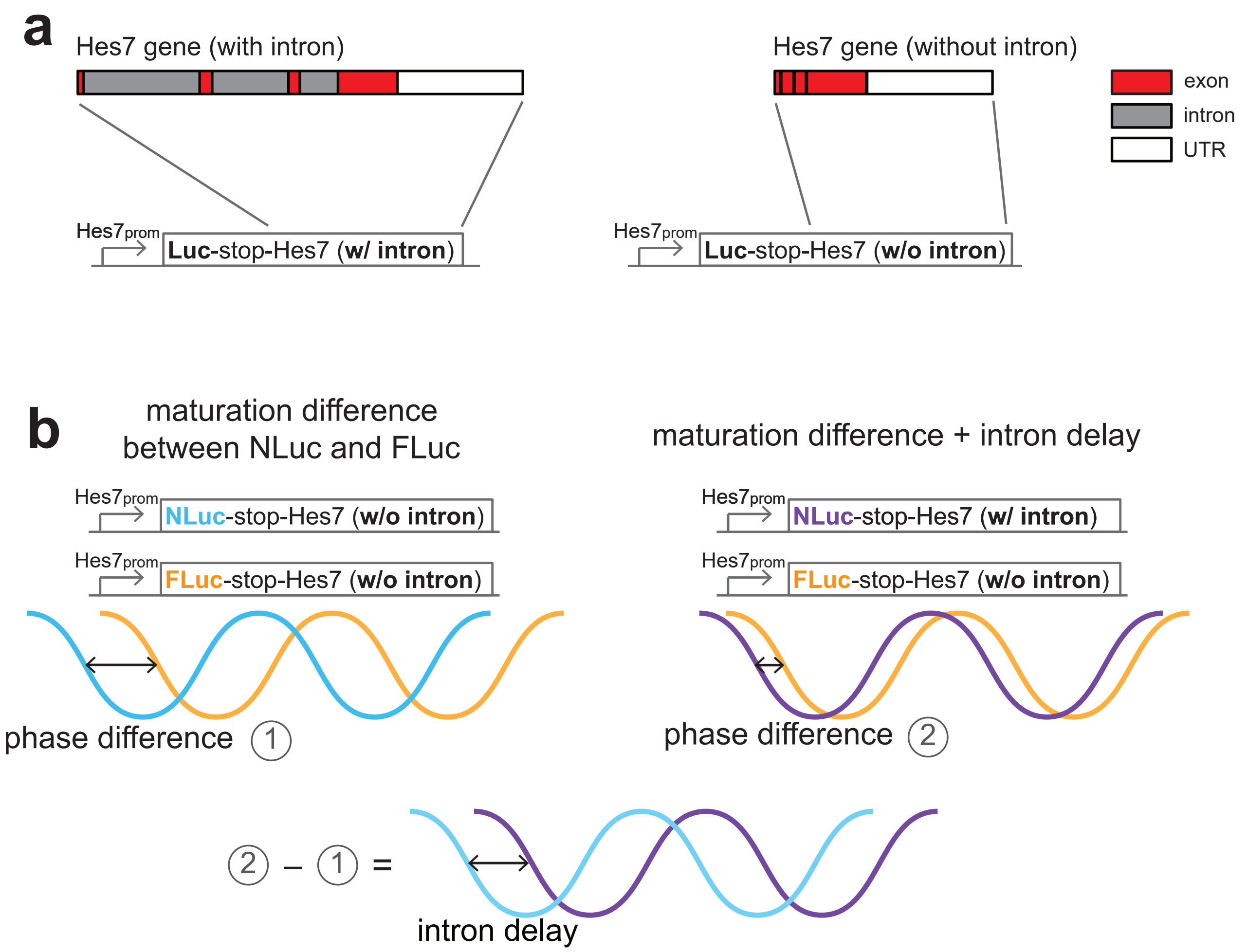

Supplementary Figure 3 Schematics of the intron delay assay

a, Constructs used for the Hes7 intron delay assay. Left panel: The *Hes7* sequence containing exons, introns, and UTR was fused after the stop codon (stop) of destabilized luciferase. Right panel: The 'without (w/o) intron' construct is the 'with (w/) intron' construct lacking introns. For the actual sequences, please refer to Matsuda et al. (2020)[25]. **b,** Schematic representation of the intron delay assay. As explained in Fig. 2f, Hes7 intron delay is defined as the oscillation phase difference between two reporters w/o and w/ *Hes7* intron sequences, namely the phase difference between NLuc-Hes7 (w/o intron) (blue) and NLuc-Hes7 (w/ intron) (purple) reporters. However, since different spectra of NLuc and FLuc are required to simultaneously monitor the oscillatory activities of two reporters, the FLuc-Hes7 (w/o intron) reporter (orange) is also required. Step 1 (left panel): To normalize the maturation/degradation time difference between NLuc and FLuc proteins, the phase difference between blue and orange lines is calculated. Step 2 (right panel): The phase difference between purple and orange lines is calculated. Step 3 (bottom panel): The intron delay (the phase difference between blue and purple lines) is calculated by subtracting the phase difference in Step 1 from that in Step 2.

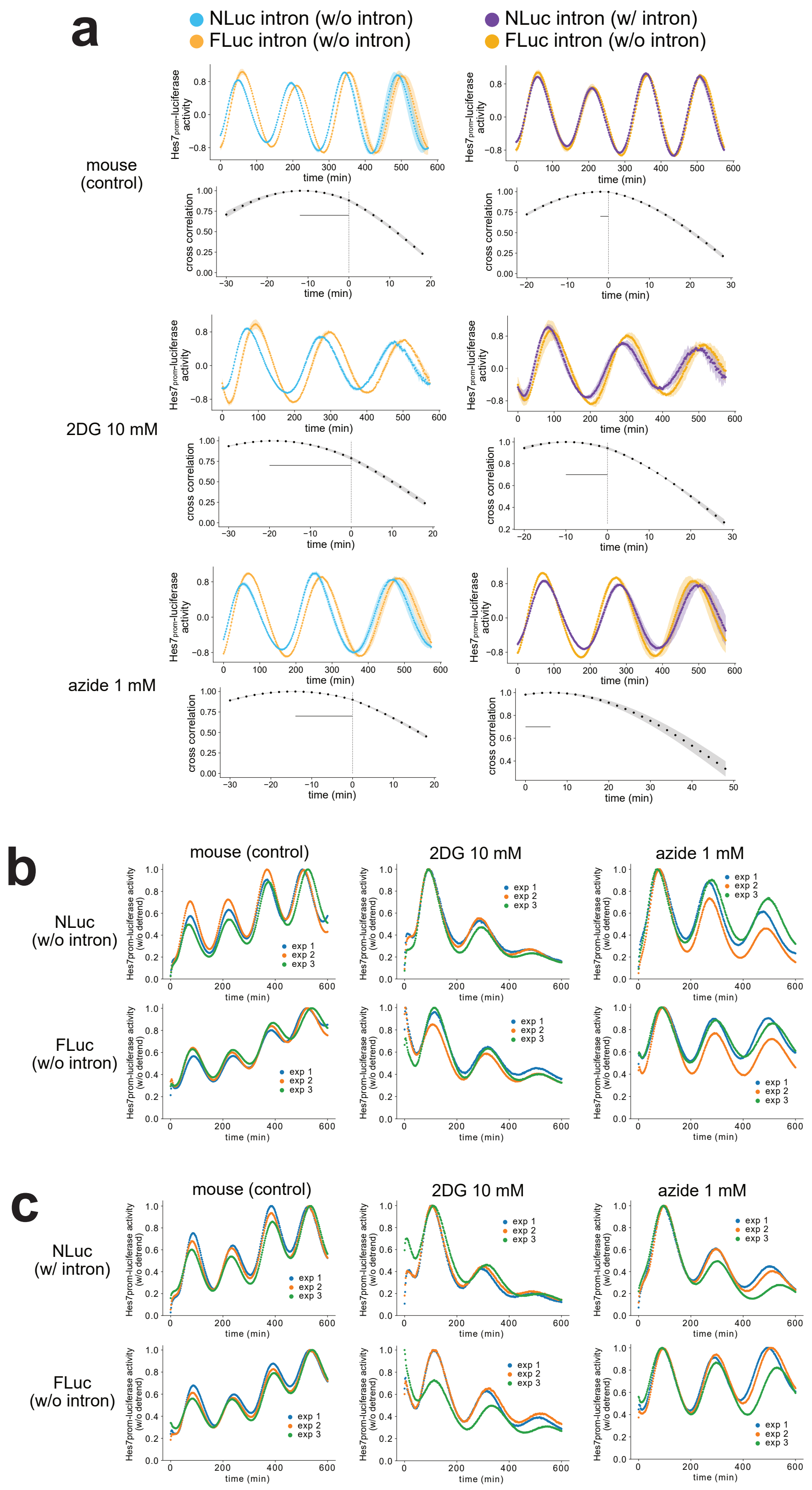

Supplementary Figure 4 Intron delay assay

a, Original data of the Hes7 intron delay assay shown in Fig. 2g. Shading indicates mean ± sd (n = 3). Top panels: Oscillatory activities of two reporters were monitored simultaneously. The signal was detrended and amplitude-normalized. Bottom panels: Cross-correlation of the two reporters. The peak of the cross-correlation was used to calculate the oscillation phase difference of the two reporters. The time 0 in a corresponds to 24 min in b and c. The intron delay (the phase difference between blue and purple lines) was estimated by subtracting the phase difference between blue and orange lines from that between purple and orange lines. **b,** Raw data of the oscillatory signals shown in a, left panels. **c,** Raw data of the oscillatory signals shown in a, right panels. **b,c,** Individual tracks from 3 independent experiments. The maximum value was set to 1. Source data are provided as a Source Data file.

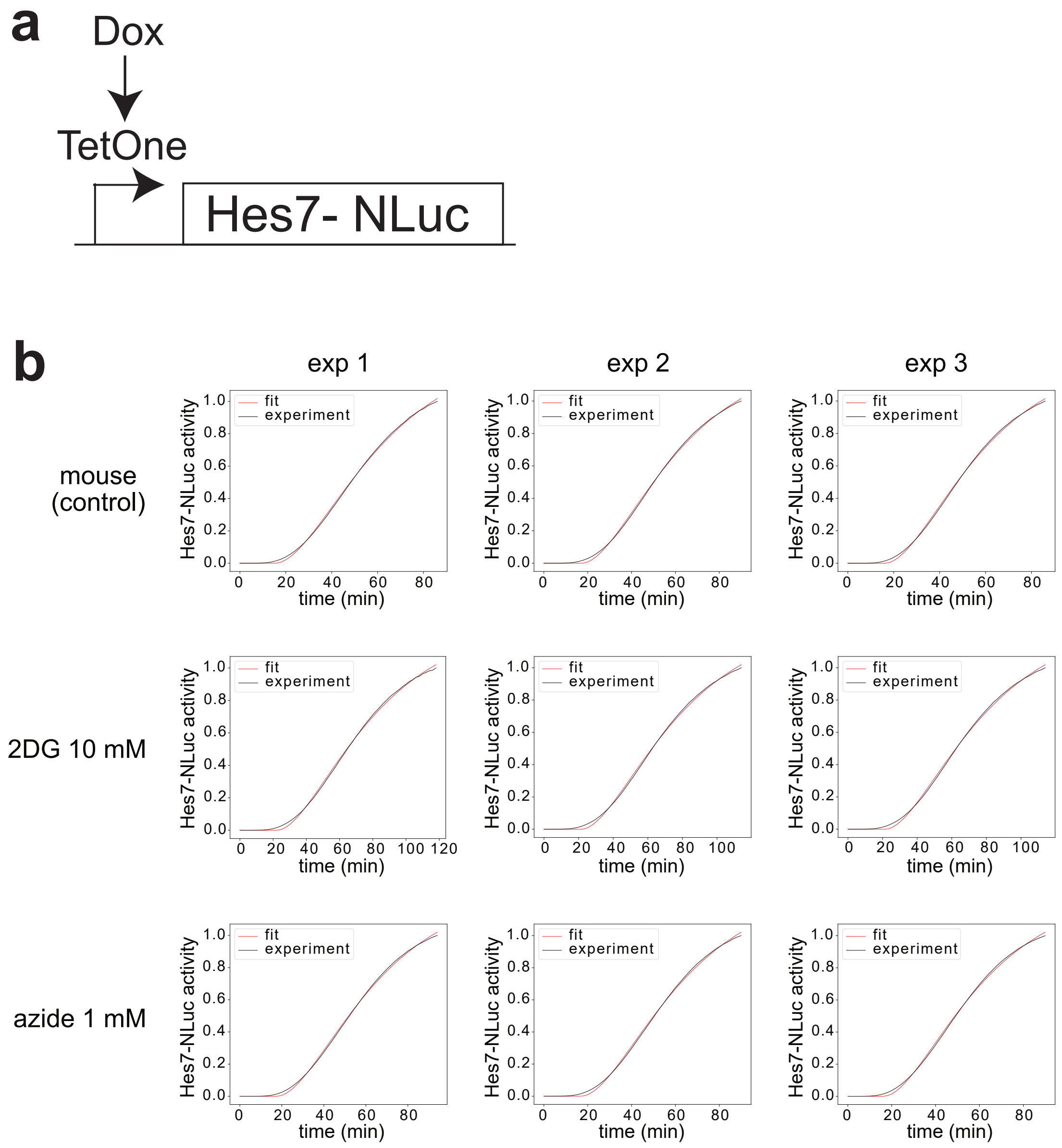

Supplementary Figure 5 Production delay assay

a, Schematic representation of the TetOne promoter-Hes7 reporter. Upon Dox addition at time 0, TetOne promoter is activated, and the transcription of Hes7-NLuc is initiated. By monitoring the onset of the Hes7-NLuc signal, the production delay is estimated. **b,** Raw data and fitting of the Hes7 production delay assay shown in Fig. 2i. The data within 2×(duration to reach the inflection point) were used for fitting, and the inflection point was determined by calculating the 2nd derivatives of the data. Source data are provided as a Source Data file.

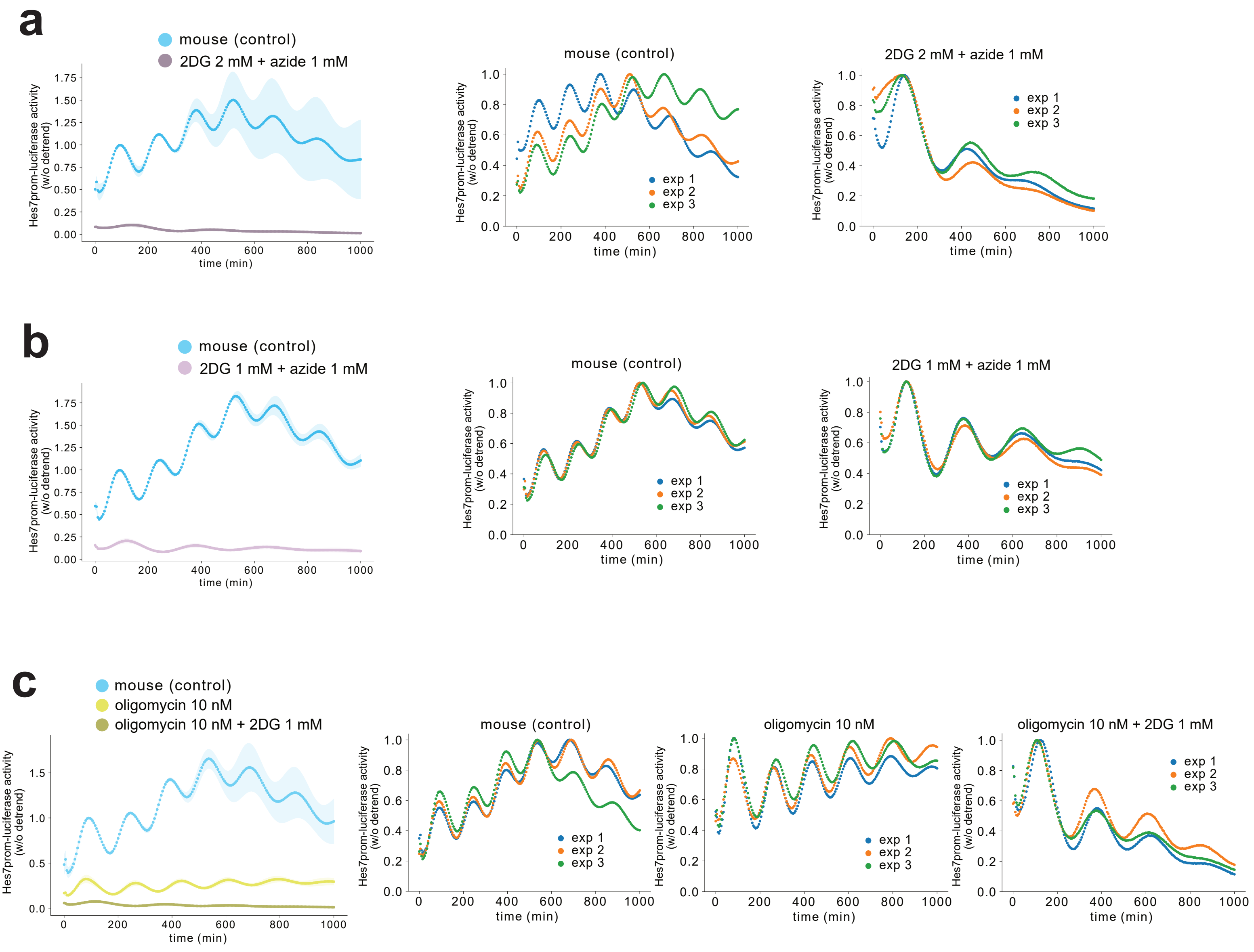

Supplementary Figure 6 Effects of double metabolic inhibitions on raw oscillatory signals

a-c, Raw data of the oscillatory signals shown in Fig. 3a,c,e. Left panels: Averaged signal. Shading indicates mean ± sd (n = 3). The first peak of the oscillatory signal of the control sample was set to 1. Right panels: Individual tracks of the left panel from 3 independent experiments. The maximum value was set to 1. Source data are provided as a Source Data file.

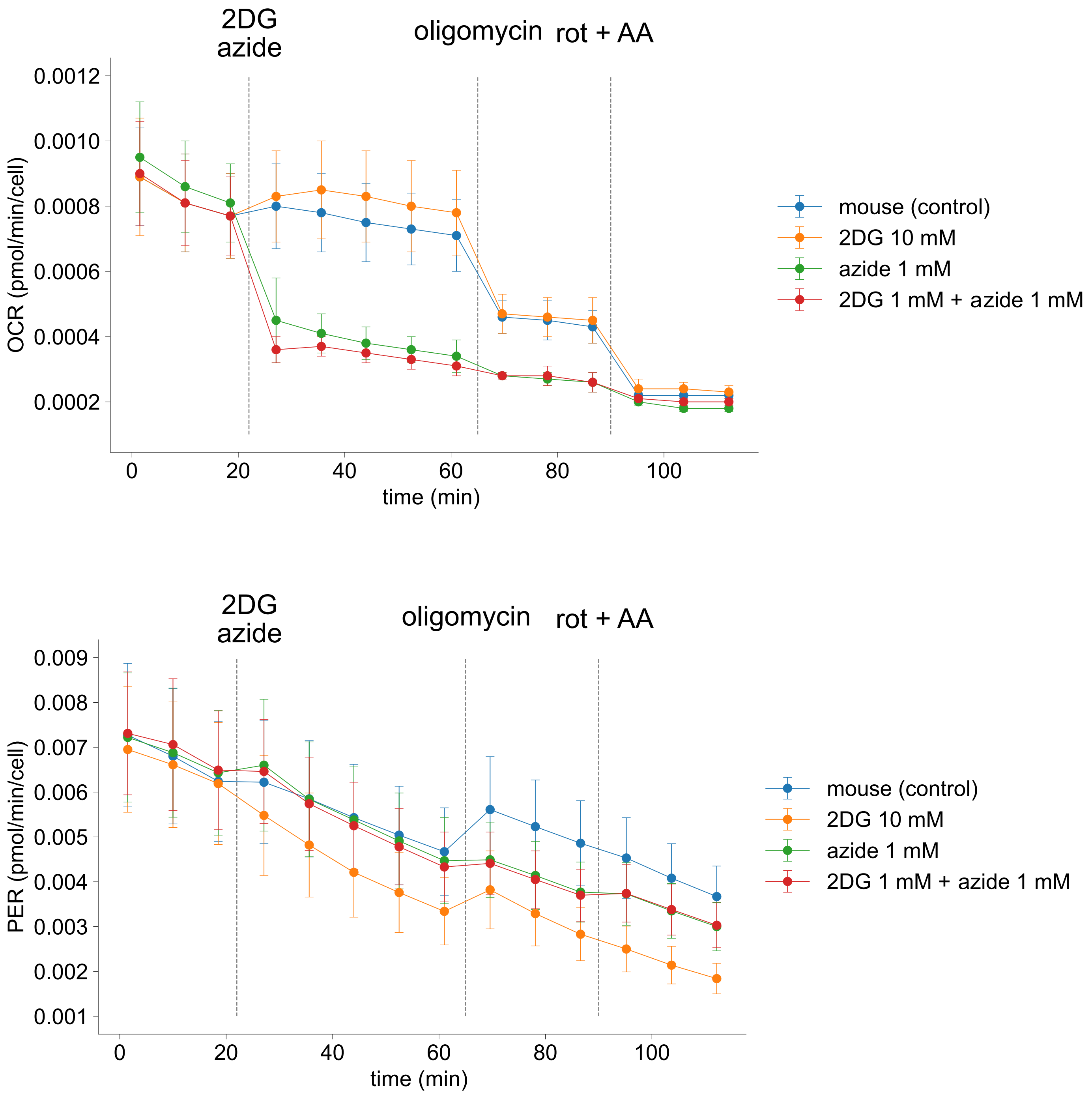

Supplementary Figure 7 Metabolic rate measurement

Raw data of the oxygen consumption rate (OCR) and proton efflux rate (PER) measured over the course of the Seahorse real-time ATP rate assay shown in Fig. 3g. Metabolic inhibitors (2DG and azide), oligomycin, and rotenone + antimycin A (rot + AA) were added at the marked time points. Error bars indicate mean ± sd. Source data are provided as a Source Data file.

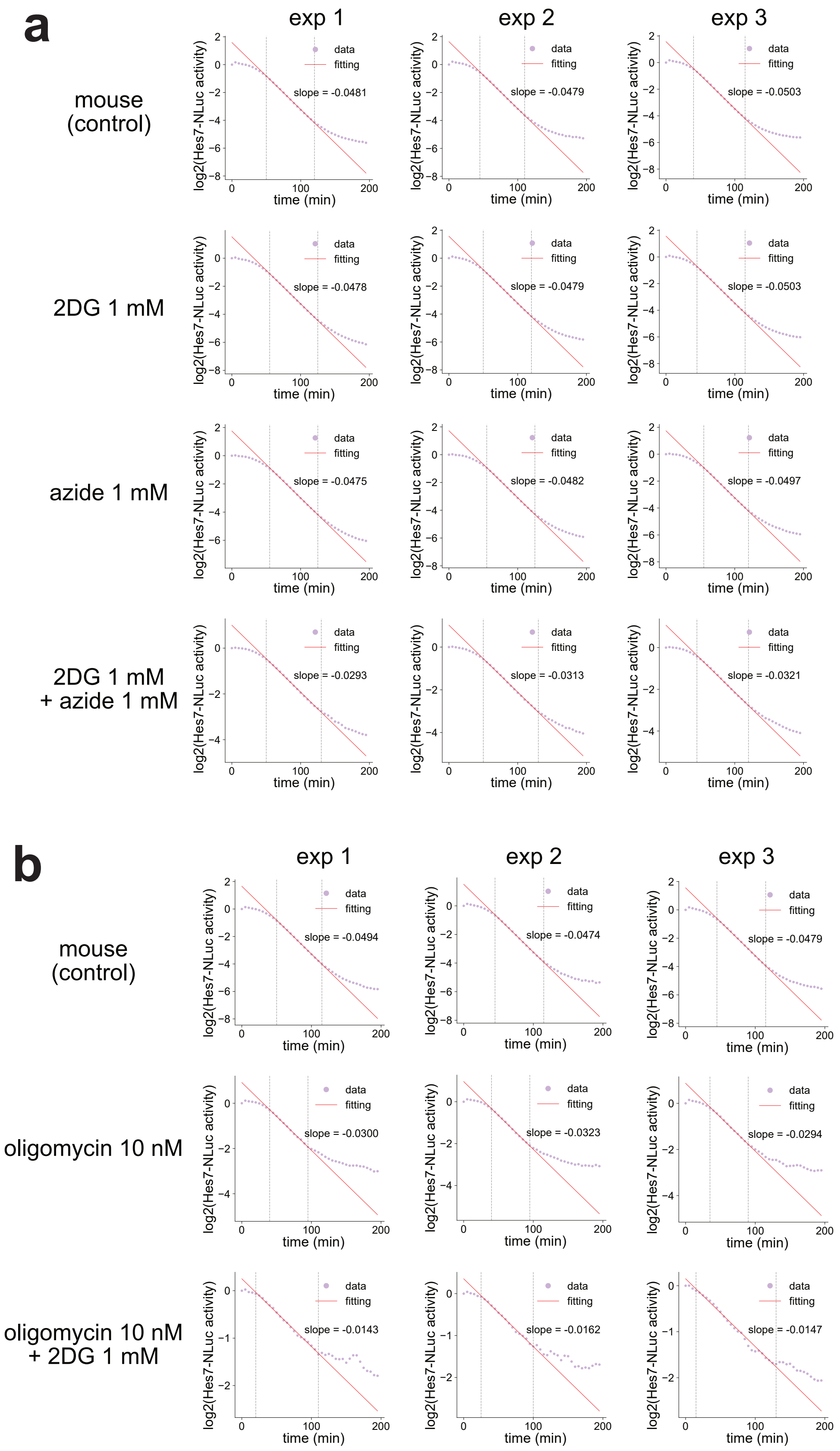

Supplementary Figure 8 Protein degradation assay with combined metabolic inhibitions

a,b, Raw data and fitting of the Hes7 protein degradation assay shown in Fig. 4b,d. Dashed lines indicate the most linear region considered by the RANSAC algorithm for the fitting. Slope of the fitted line is shown, and it was converted to the half-life using the equation: half-life = -1/slope. Source data are provided as a Source Data file.

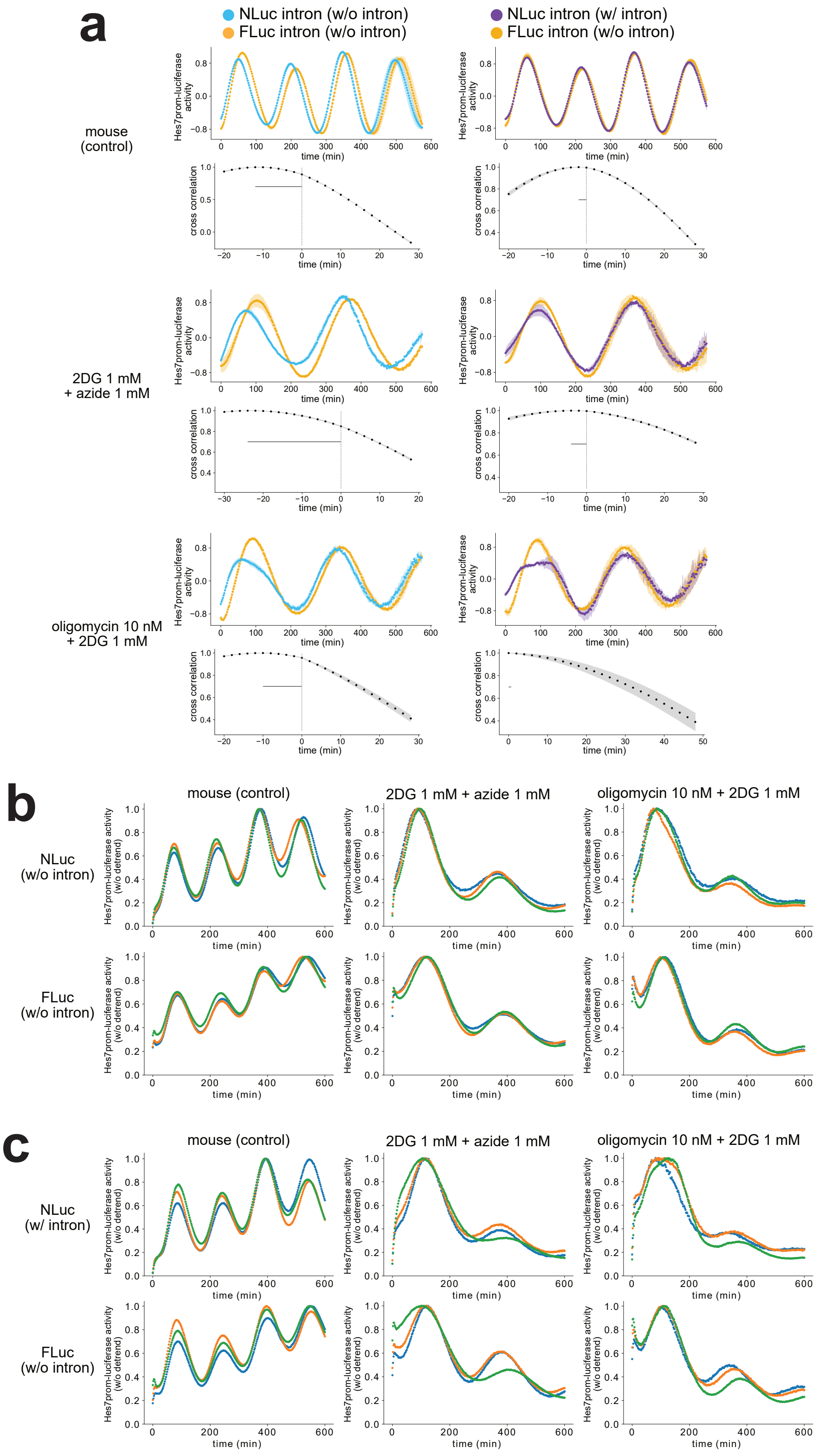

Supplementary Figure 9 Intron delay assay with combined metabolic inhibitions

a, Original data of the Hes7 intron delay assay shown in Fig. 4e. Shading indicates mean ± sd (n = 3). Top panels: Oscillatory activities of two reporters were monitored simultaneously. The signal was detrended and amplitude-normalized. Bottom panels: Cross-correlation of the two reporters. The peak of the cross-correlation was used to calculate the oscillation phase difference of the two reporters. The time 0 in a corresponds to 24 min in b and c. The intron delay (the phase difference between blue and purple lines) was estimated by subtracting the phase difference between blue and orange lines from that between purple and orange lines. **b,** Raw data of the oscillatory signals shown in a, left panels. **c,** Raw data of the oscillatory signals shown in a, right panels. **b,c,** Individual tracks from 3 independent experiments. The maximum value was set to 1. Source data are provided as a Source Data file.

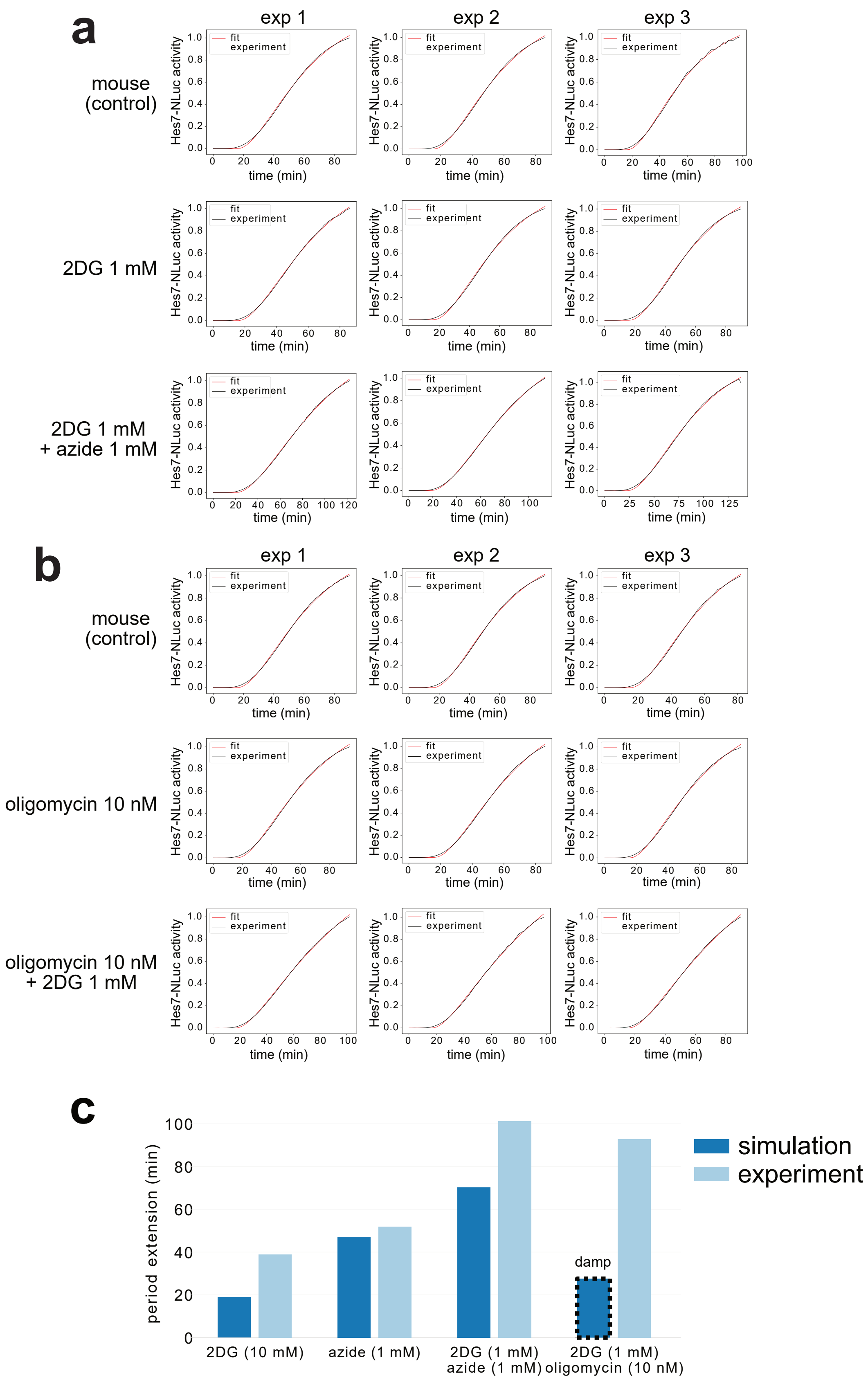

Supplementary Figure 10 Production delay assay and period simulation with combined metabolic inhibitions **a,b**, Raw data and fitting of the Hes7 production delay assay shown in Fig. 4g,i. The data within 2×(duration to reach the inflection point) were used for fitting, and the inflection point was determined by calculating the 2nd derivatives of the data. **c,** Simulated segmentation clock periods based on the degradation rates and delays measured in mouse PSM cells under indicated metabolic inhibitions. The extension of the oscillation period compared to the control is shown. Note that the simulation of the 2DG + oligomycin condition resulted in damped oscillation. Experimentally measured periods are from Fig. 2c; 3b,f. Source data are provided as a Source Data file.

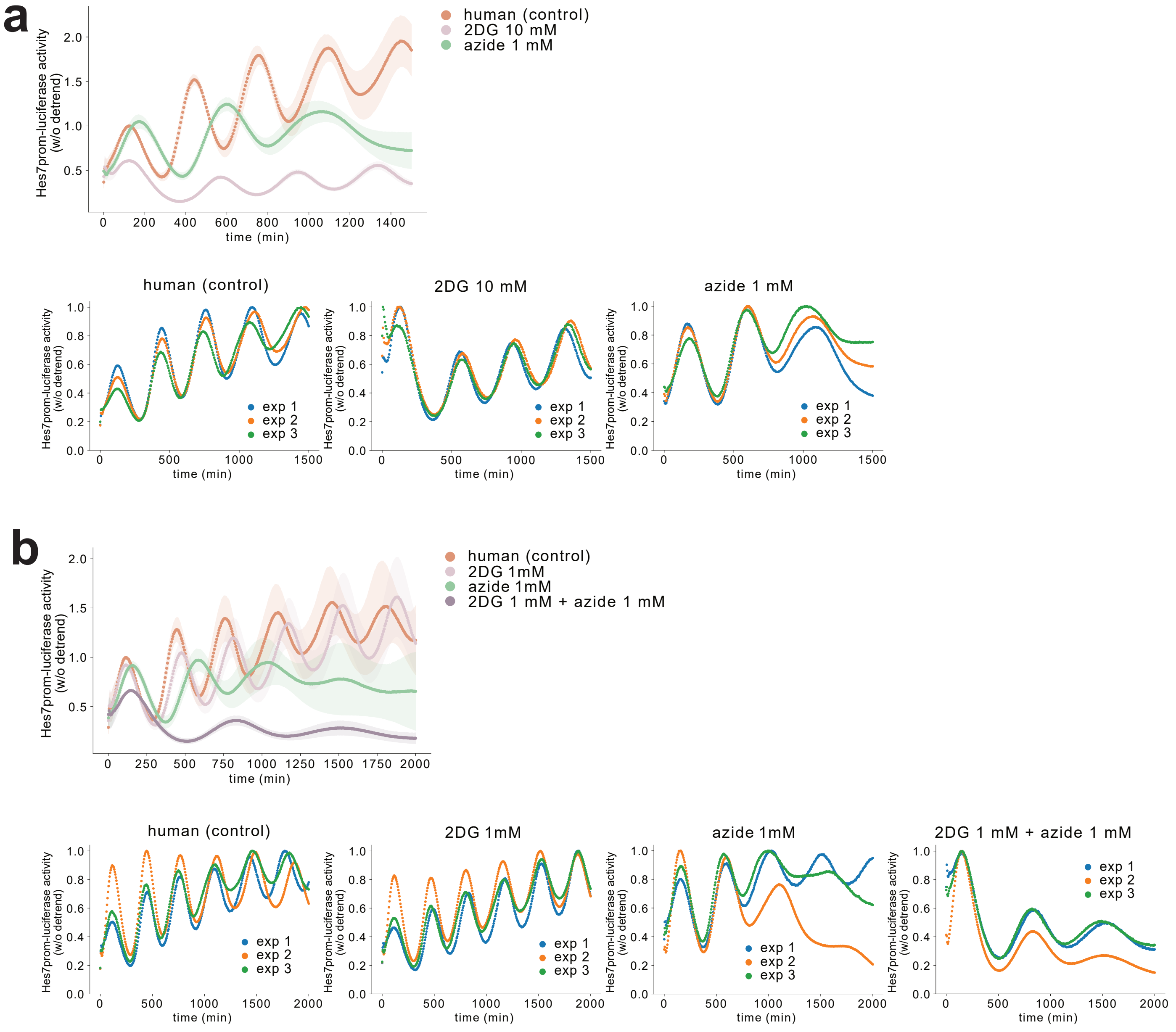

Supplementary Figure 11 Effects of metabolic inhibitions on oscillatory signals in human PSM cells

a,b, Raw data of the oscillatory signals shown in Fig. 5a,c. Top panels: Averaged signal. Shading indicates mean ± sd (n = 3). The first peak of the oscillatory signal of the control sample was set to 1. Bottom panels**:** Individual tracks of the top panel from 3 independent experiments. The maximum value was set to 1. Source data are provided as a Source Data file.

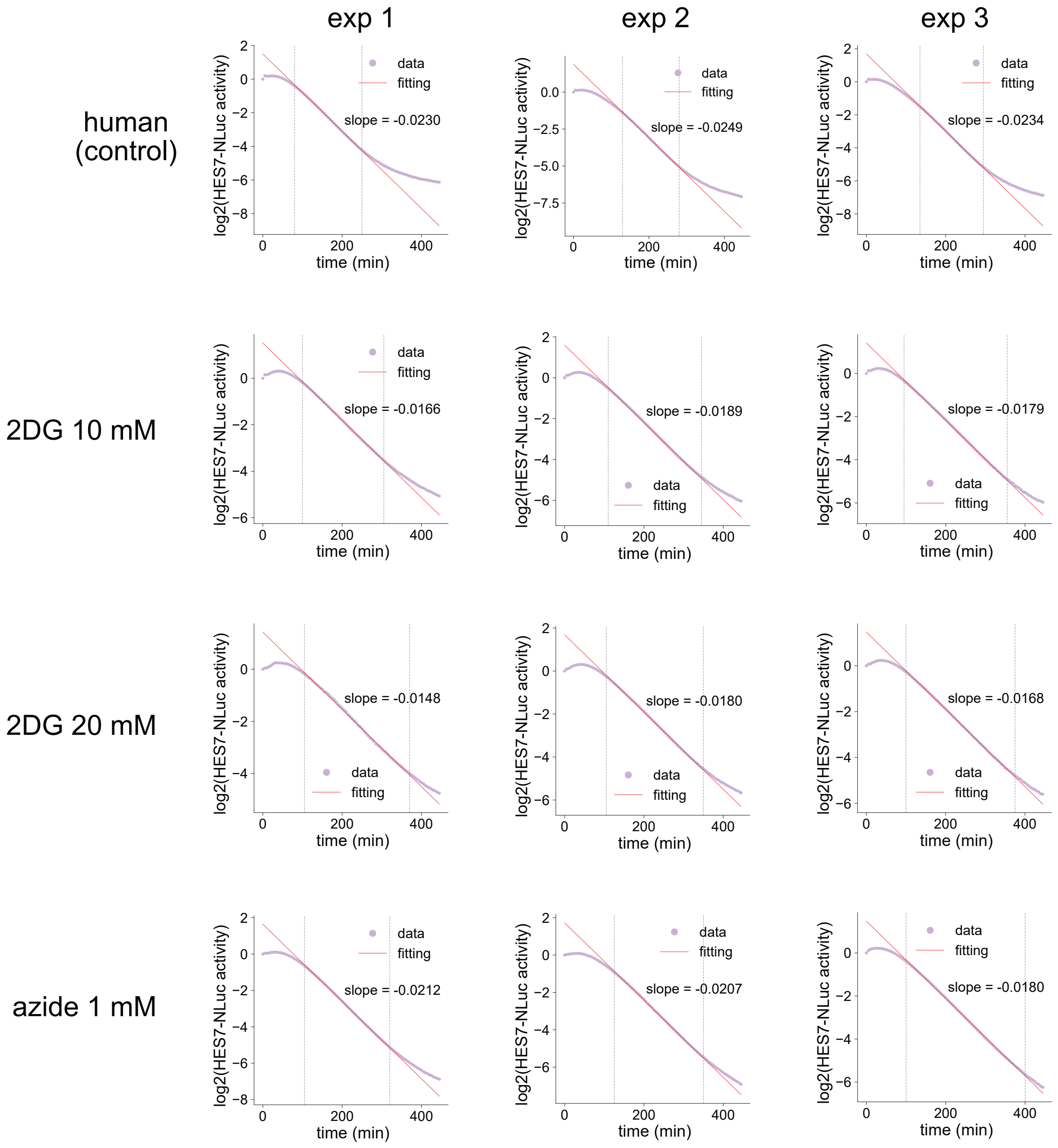

Supplementary Figure 12 Protein degradation assay in human PSM cells

Raw data and fitting of the HES7 protein degradation assay shown in Fig. 5f. Dashed lines indicate the most linear region considered by the RANSAC algorithm for the fitting. Slope of the fitted line is shown, and it was converted to the half-life using the equation: half-life = -1/slope. Source data are provided as a Source Data file.

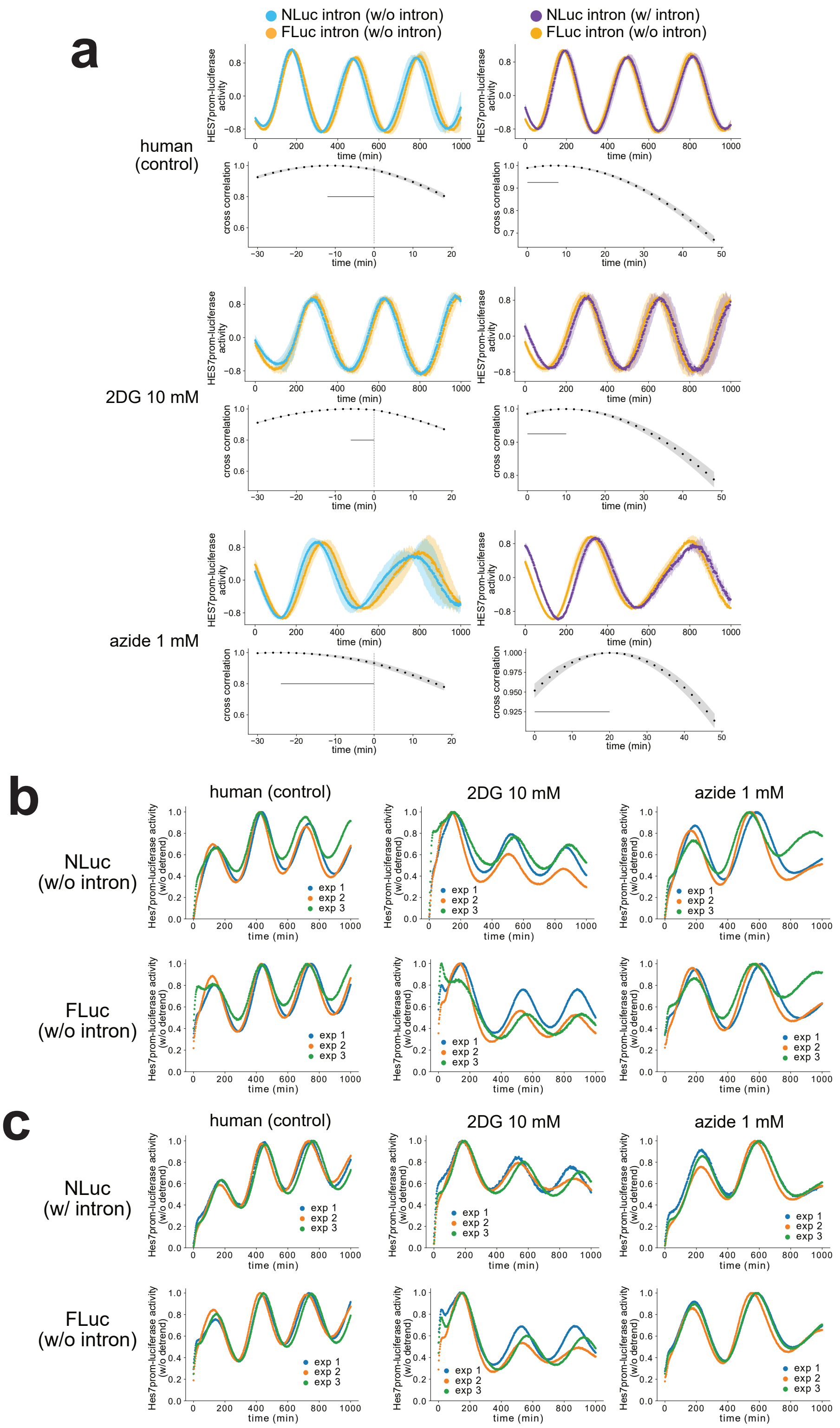

Supplementary Figure 13 Intron delay assay in human PSM cells

a, Original data of the Hes7 intron delay assay shown in Fig. 5g. Shading indicates mean ± sd (n = 3). Top panels: Oscillatory activities of two reporters were monitored simultaneously. The signal was detrended and amplitude-normalized. Bottom panels: Cross-correlation of the two reporters. The peak of the cross-correlation was used to calculate the oscillation phase difference of the two reporters. The time 0 in a corresponds to 250 min in b and c. The intron delay (the phase difference between blue and purple lines) was estimated by subtracting the phase difference between blue and orange lines from that between purple and orange lines. **b,** Raw data of the oscillatory signals shown in a, left panels. **c,** Raw data of the oscillatory signals shown in a, right panels. **b,c,** Individual tracks from 3 independent experiments. The maximum value was set to 1. Source data are provided as a Source Data file.

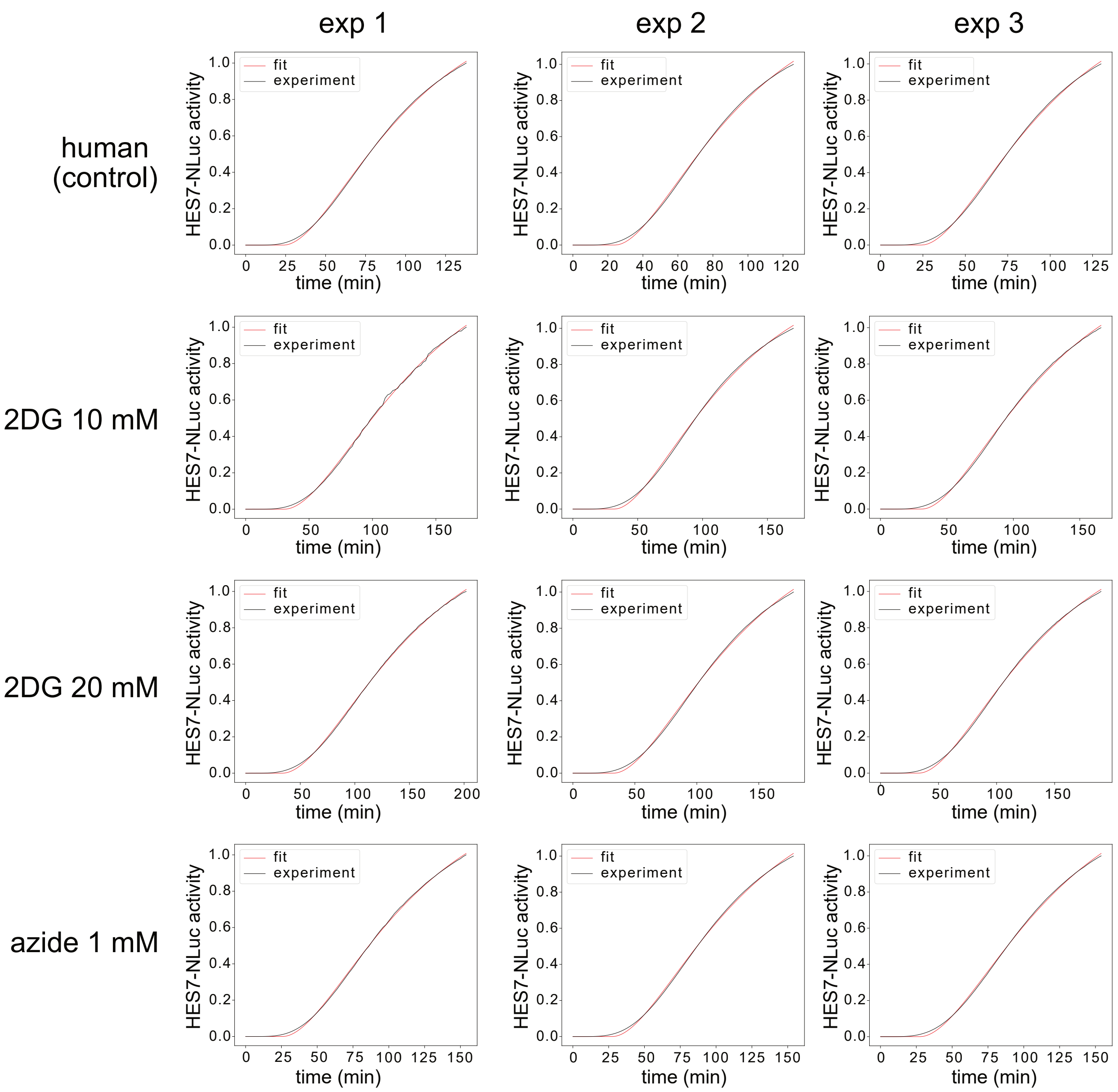

Supplementary Figure 14 Production delay assay in human PSM cells

Raw data and fitting of the HES7 production delay assay shown in Fig. 5i. The data within 2×(duration to reach the inflection point) were used for fitting, and the inflection point was determined by calculating the 2nd derivatives of the data. Source data are provided as a Source Data file.

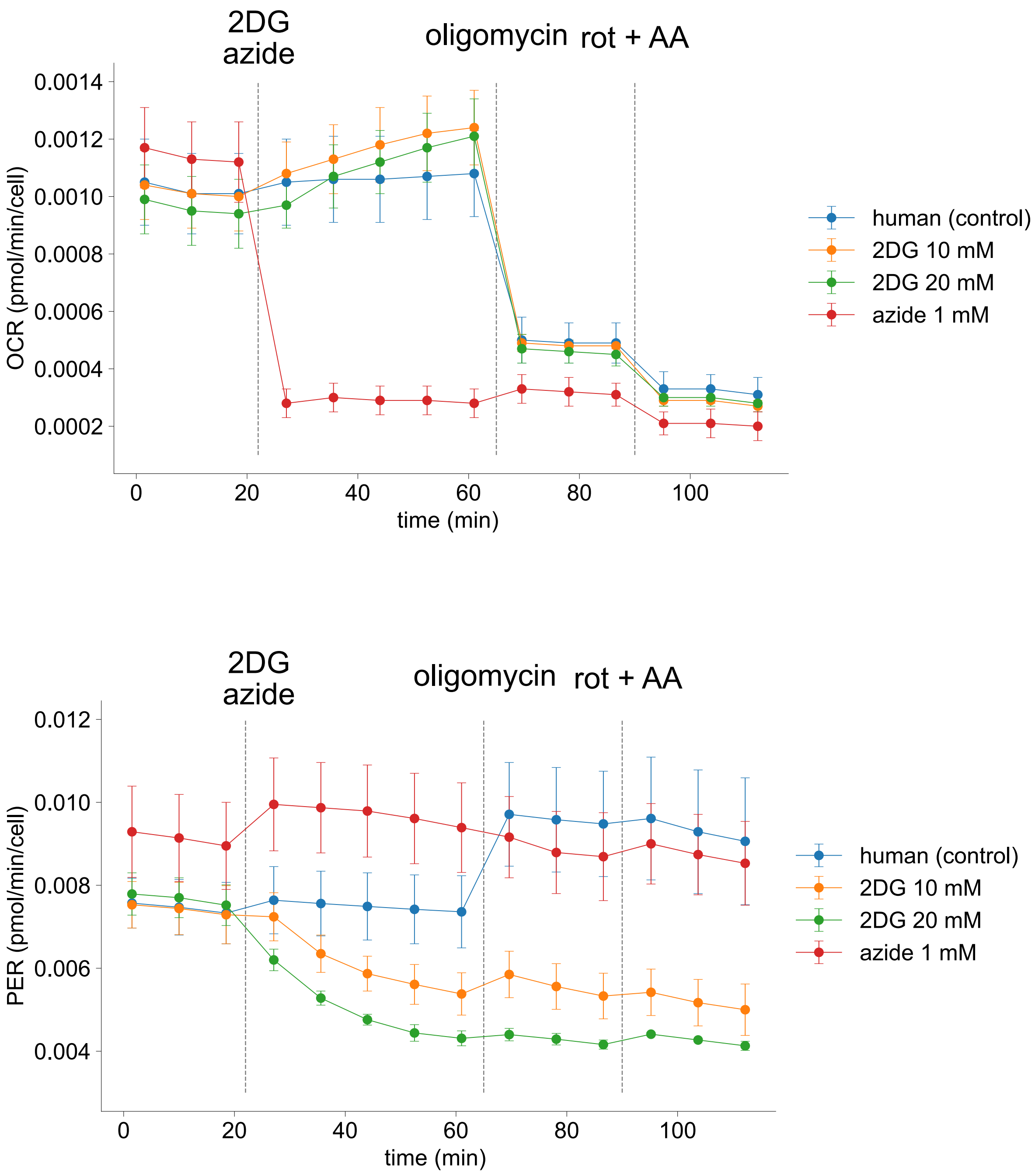

Supplementary Figure 15 Metabolic rate measurement in human PSM cells

Raw data of the oxygen consumption rate (OCR) and proton efflux rate (PER) measured over the course of the Seahorse real-time ATP rate assay shown in Fig. 5j. Metabolic inhibitors (2DG and azide), oligomycin, and rotenone + antimycin A (rot + AA) were added at the marked time points. Error bars indicate mean ± sd. Source data are provided as a Source Data file.

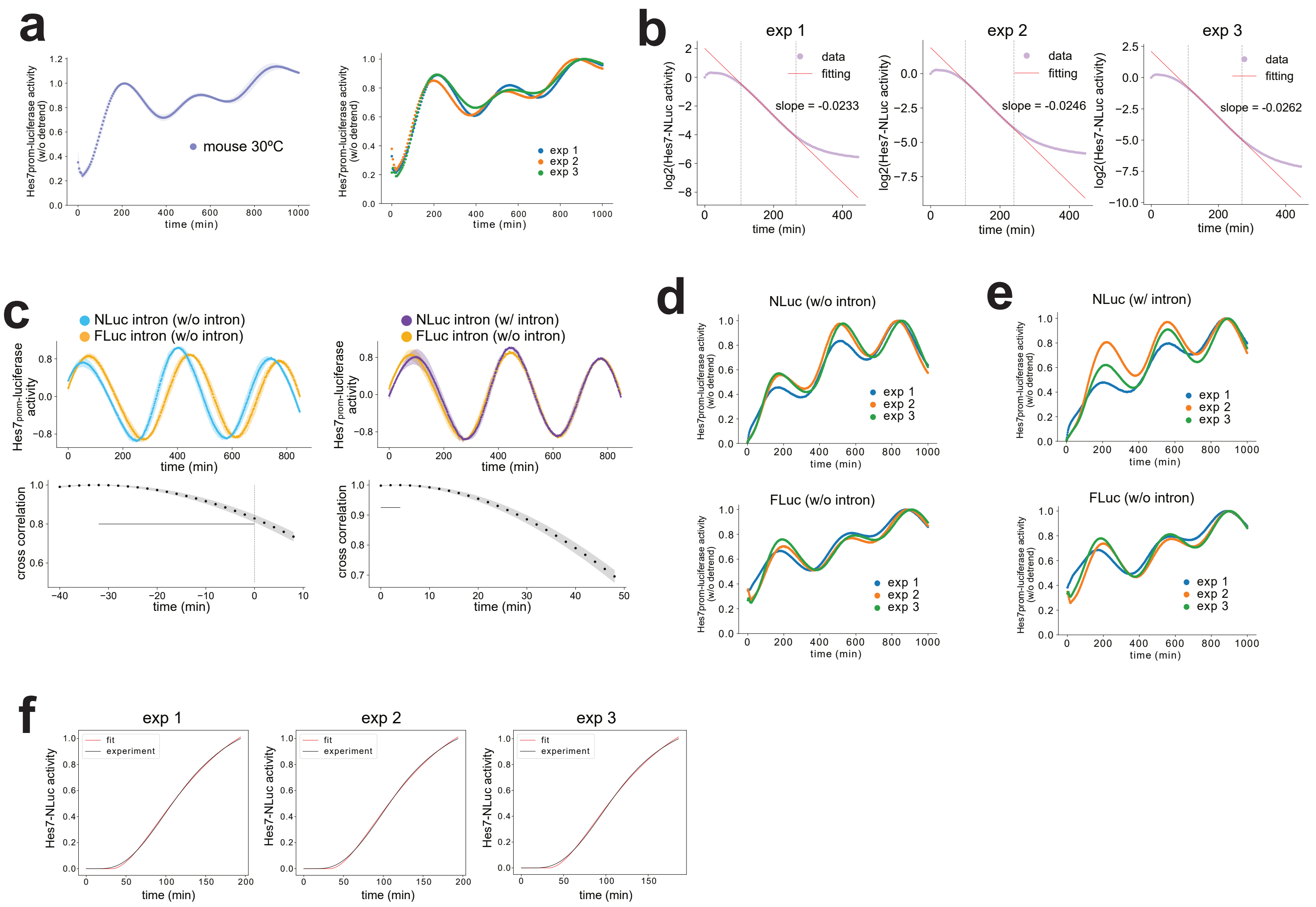

Supplementary Figure 16 Temperature change

Mouse PSM cells were incubated at 30°C instead of the standard 37°C for each assay. **a,** Raw data of the oscillatory signals shown in Fig. 6a. Left panel: Averaged signal. Shading indicates mean ± sd (n = 3). The first peak of the oscillatory signal of the control sample was set to 1. Right panel**:** Individual tracks of the left panel from 3 independent experiments. The maximum value was set to 1. **b,** Raw data and fitting of the Hes7 protein degradation assay shown in Fig. 6d. Dashed lines indicate the most linear region considered by the RANSAC algorithm for the fitting. Slope of the fitted line is shown, and it was converted to the half-life using the equation: half-life = -1/slope. **c,** Original data of the Hes7 intron delay assay shown in Fig. 6e. Shading indicates mean ± sd (n = 3). Top panels: Oscillatory activities of two reporters were monitored simultaneously. The signal was detrended and amplitude-normalized. Bottom panels: Cross-correlation of the two reporters. The peak of the cross-correlation was used to calculate the oscillation phase difference of the two reporters. The time 0 in a corresponds to 100 min in b and c. The intron delay (the phase difference between blue and purple lines) was estimated by subtracting the phase difference between blue and orange lines from that between purple and orange lines. **d,** Raw data of the oscillatory signals shown in c, left panel. **e,** Raw data of the oscillatory signals shown in c, right panel. **d,e,** Individual tracks from 3 independent experiments. The maximum value was set to 1. **f,** Raw data and fitting of the Hes7 production delay assay shown in Fig. 6g. The data within 2×(duration to reach the inflection point) were used for fitting, and the inflection point was determined by calculating the 2nd derivatives of the data. Source data are provided as a Source Data file.
